# Variations in Fibrinogen-like 1 (*FGL1*) Gene Locus as a Genetic Marker Related to Fat Deposition Based on Pig Model and Liver RNA-Seq Data

**DOI:** 10.3390/genes13081419

**Published:** 2022-08-09

**Authors:** Katarzyna Piórkowska, Kacper Żukowski, Katarzyna Ropka-Molik, Mirosław Tyra

**Affiliations:** 1 Department of Animal Molecular Biology, National Research Institute of Animal Production, Krakowska 1, 32-083 Balice, Poland; 2 Department of Cattle Breeding, National Research Institute of Animal Production, Krakowska 1, 32-083 Balice, Poland; 3 Department of Pig Breeding, National Research Institute of Animal Production, Krakowska 1, 32-083 Balice, Poland

**Keywords:** fibrinogen-like 1, *FGL1*, pig, fat deposition, selective markers

## Abstract

The goal of this study was to evaluate the effects of mutations in the *FGL1* gene associated with pig productive traits to enrich the genetic marker pool for further selection and to support the studies on *FGL1* in the context of the fat deposition (FD) process. The variant calling and χ^2^ analyses of liver RNA-seq data were used to indicate genetic markers. *FGL1* mutations were genotyped in the Złotnicka White (n = 72), Polish Large White (n = 208), Duroc (n = 72), Polish Landrace (PL) (n = 292), and Puławska (n = 178) pig breeds. An association study was performed using a general linear model (GLM) implemented in SAS^®^ software. More than 50 crucial mutations were identified in the *FGL1* gene. The association study showed a significant effect of the *FGL1* on intramuscular fat (IMF), loin eye area, backfat thickness at the lumbar, ham mass (*p* = 0.0374), meat percentage (*p* = 0.0205), and loin fat (*p* = 0.0003). Alternate homozygotes and heterozygotes were found in the PL and Duroc, confirming the selective potential for these populations. Our study supports the theory that liver *FGL1* is involved in the FD process. Moreover, since fat is the major determinant of flavor development in meat, the *FGL1* rs340465447_A allele can be used as a target in pig selection focused on elevated fat levels.

## 1. Introduction

Next-generation sequencing (NGS) technology is currently widely applied to predict processes associated with fat deposition and obesity-related events [1,2]. Besides whole-genome sequencing (WGS) and genome-wide association studies (GWASs) [3], RNA sequencing delivers valuable information about polymorphic sites located in transcripts [4] to develop selective markers related to given traits. However, the main target of RNA-seq analysis is to estimate transcript levels, including ncRNA molecules such as lncRNA and miRNA [5]. Variant calling analysis based on transcriptome sequencing results could be an alternative way to identify gene mutations associated with the fat deposition (FD) process. Although, this type of analysis requires more detailed calculations than SNP identification-based WGS data [6]. Nevertheless, identifying mutations within RNA-seq data is the additive value because it requires only an extra in silico analysis.

Fibrinogen-like protein 1 (*FGL1*) is a member of the fibrinogen family mainly synthesized in the liver [7]. It has been demonstrated that increased *FGL1* expression is related to the regeneration of the liver organ by stimulating 3H-thymidine uptake leading to hepatocyte proliferation [8]. *FGL1* is also expressed in brown adipose tissue (BAT) and during liver injury; expression is also upregulated in this tissue, which suggests cross-talk between an injured liver and bAT. Consistently, a study of Fgl1-deficient mice showed global metabolic disorders, namely *FGL1*-null mice were heavier, had abnormal plasma lipid profiles, fasting hyperglycemia with enhanced gluconeogenesis, and exhibited differences in white and BAT morphology regarding wild types [9]. The authors implied that the structural similarity of Fgl1 to angiopoietin factors regulating lipid metabolism showed that *FGL1* likely plays a crucial role in lipid metabolism and liver regeneration. On the other hand, *FGL1* expression has been examined in gastric cancer (GC) tissue [7], where *FGL1* promotes GC proliferation, and patient overall survival time with increased *FGL1* expression was significantly shorter. Therefore, the *FGL1* gene has been proposed as a predictor in GC patients and a target for treating this cancer type. Wang et al. [10] showed that Fgl1 is a major ligand for immune inhibitory receptor lymphocyte-activation gene 3 (LAG-3) and inhibits antigen-specific T cell activation. According to these findings, elevated *FGL1* expression in the plasma of cancer patients is related to poor prognosis.

Pigs are a highly suited animal model for predicting FD events due to many convergences with the human body, such as the size of particular organs [11], body fat distribution, and similar metabolism [12].

In the present study, Złotnicka White (ZW) pigs were used as an animal model in the context of FD processes. ZW is a Polish indigenous breed included in the Polish Animal Genetic Resource Conservation Program [13] and has never been under selection pressure, thus retaining a high meat quality [14]. In addition, ZW pigs are differentiated in terms of fat deposition in the carcass; therefore, it was possible to select two groups to represent low and high-FD values. Variant calling analysis based on liver RNA-seq data identified, in a cheaper manner than GWAS, numerous polymorphic sites in transcripts, and the χ^2^ test pinpointed mutations characterized by significantly different allele distributions between the FD pig groups. Following this work scheme, we found *FGL1* variants highly interesting regarding the possible application in the selection process.

## 2. Materials and Methods

Samples were collected after slathering in the Pig Test Station (PTS, National Research Institute of Animal Production). Following carcass evaluation, the meat was intended for sale and consumption. Ethical review and approval were waived for this study due to a non-invasive method of collecting. All conducted research was approved by the Approving Experiment Committee of the National Research Institute of Animal Production (Krakow, Poland) according to the Polish Act on the Protection of Animals Used for Scientific or Educational Purposes of 15 January 2015, which implements Directive 2010/63/EU of the European Parliament and the Council of 22 September 2010 on the protection of animals used for scientific purposes. Moreover, all procedures followed the guidelines and regulations of the Local Krakow Ethics Committee for Experiments with Animals.

### 2.1. Animals and Methods

Female pigs of Złotnicka White were used in RNA-seq (n = 16) and association (n = 72) analyses. The animals with an initial weight of 30 kg were delivered to the Pig Test Station (PTS) of the National Research Institute of Animal Production from different farms. The animals were maintained under the same environmental and diet conditions according to a local procedure [15], feeding *ad libitum* to the final weight of 100 ± 2.5 kg. During pig maintenance at the PTS, growth performance such as feed intake, feed conversion, and daily gain were measured. After slaughter and detailed dissection, body composition traits were measured, including meat percentage, carcass yield, weight of the most significant cuts such as ham and loin, backfat thickness, and numerous fat-related traits, including visceral and subcutaneous fat content and meat quality traits such as intramuscular fat (IMF), pH, meat color, and myowater exudation [16].

Liver samples (n = 72) were collected within 20 min after dissection and stabilized in RNAlater™ Stabilization Solution (Invitrogen, Waltham, MA, USA), and then frozen at −20 °C. Next, two pig groups were selected based on fat-level measurements performed 24 h after slaughter. The RNA-seq experiment included 16 ZW pigs, with 8 representing high-fat deposition (HFD, n = 8) and low (LFD, n = 8) values.

Moreover, *FGL1* variant frequency was estimated in Polish Large White (PLW) (n = 208), Polish Landrace (PL) (n = 292), Puławska (n = 178), and Duroc (n = 72) pigs that are still active in the Polish breeding. Animal samples for DNA isolation were collected as blood or hair follicles and were banked in the National Research Institute of Animal Production as part of other research projects.

### 2.2. Library Construction, Sequencing, and Aligning Raw Reads to the Pig Transcriptome

As described previously by Piórkowska et al. [17], RNA was isolated using a PureLink™ RNA Mini Kit (Invitrogen, Waltham, MA, USA). Its quality and quantity were calculated using TapeStatio2200 (RNA tape, Agilent, Santa Clara, CA, USA), and the RIN parameters were over 7.5 value. The cDNA libraries were prepared using a TruSeq RNA Sample Preparation Kit v2 (Illumina, San Diego, CA, USA), with a unique barcode for each sample. The quality and quantity of the cDNA libraries were assessed by the Qubit Fluorimeter (Invitrogen, Waltham, MA, USA) and TapeStation 2200 system (D1000 tape, Agilent, Santa Clara, CA, USA). The final concentration of cDNA libraries’ was normalized to 10 nM, and they were pooled. Transcriptome sequencing was performed in 150PE cycles on the HiSeq 3000 System (Illumina, San Diego, CA, USA), employing commissioned sequencing in Admera Health Biopharma Services. Aligning of raw reads to the pig transcriptome was performed according to Piórkowska et al. [17].

### 2.3. Transcript Variant Identification

Picard tools and GATK v. 4.1.9 [18] were used to split reads containing Ns in their CIGAR string and for the base quality score recalibration, indel realignment, removal of duplicates, and finally to identify SNPs and indels. The filtering parameters were selected according to the Best Practices workflow [6,19]. Mutation sites for annotation and prediction were analyzed by SnpEff v. 4.1b [20] and the Variant Effect Predictor (Ensembl) [21]. Differences in allele distribution of mutations identified by RNA-seq using the χ^2^ test (corrected p-value at false discovery rate (FDR) ≤ 0.05) between the HFD and LFD pigs were estimated. The FDR correction was conducted using the R_stats_ base procedure and default parameters. In the χ^2^ test, it was expected that allele/genotypes would have normal according to Gaussian distribution. The procedure in R was based on Benjamin and Hochberg [22]. Functional analysis for proteins encoded by identified genes was performed by the STRING 11.0b tool (https://string-db.org/).

Four of the most significant *FGL1* variants located in the 3′UTR region, according to the χ^2^ test, were validated by Sanger sequencing of DNA. The primers for sequencing are shown in Table S1. New indel mutations identified in the *FGL1* gene were submitted to GenBank with access number MW827172 (NCBI database). miRBase v.22 and mirPath v3.0 (Diana) tools we used to analyze significant SNPs and indels identified in the 3′UTR regions for miRNA binding and miRNA functional analysis, respectively.

Linkage disequilibrium was estimated with analysis based on Barrett et al. [23] method.

### 2.4. FGL1 Genotyping, Frequency Estimation, and Statistical Analyses

For *FGL1* 3′UTR genotyping, PCR-RFLP, Sanger sequencing, and PCR-ACRS methods were designed. The primers and restriction enzymes are shown in Table S1. *FGL1* frequency was estimated for ZW (sows), PLW (boar and sows), PL (boars), Puławska (boars and sows), and Duroc (boars) pigs. Besides ZW, all individuals used in the study are still active in Polish breeding. The examination of *FGL1* variants frequency allowed us to establish the selective potential targeted to increase organoleptic qualities of pork lost in the previous intense breeding.

The differences in fat deposition traits and pig phenotypes, including 16 ZW pigs used previously in RNA sequencing analysis, were calculated between HFD and LFD using the ANOVA procedure (SAS v. 8.02) with a post-hoc Duncan test (SAS Enterprise).

Association analysis was performed, including 72 ZW pigs using the GLM procedure (SAS v. 8.02). Additionally, severely affected pig phenotype *RYR1* mutation was genotyped in the ZW population. All individuals included in the analysis were free of the *RYR1* alternate allele [24].

The general linear model (GLM) (SAS Enterprise v. 7.1 with default settings; SAS Institute, Cary, NC, USA) was used for statistical analyses. The linear model for fixed analysis was:

Y_ijkl_ = μ + d_i_ + b_j_ + α(x_ijk_) + e_ijkl_,

where: Y_ijk_—observation, µ—overall mean, d_i_—fixed effect of genotype group, b_j_—fixed effect of the breed, α(_xijk_)—covariate for weight of the right side of the carcass, and e_ijkl_—random error. The GLM model for analysis within breeds omitted b_j_—fixed effect of breed. The ratio between investigated pigs and their mothers (sows) was 1.7, and the ratio between investigated pigs and their fathers (sires) was 2.28; therefore, these factors were not included in the statistical model. During the whole year, the animals were maintained in the pig house at the same temperature and humidity conditions and with the same feeding; therefore, the model did not account for the effect of slaughter season. The least-square means (LSM) method was used for the determination of significant differences between genotype groups. The differences in phenotype in particular genotype groups are presented as LSM ± SE.

Additive and dominance effects were calculated for a total of 72 pigs. Additive and dominance effects were calculated using the regression (REG) procedure (SAS v. 8.02). The additive effect was denoted as −1 and 1 for genotypes AA and GG, respectively. The dominance effects are represented as −1 for AG heterozygotes and 1 for both homozygotes.

## 3. Results

### 3.1. Animals Used in the RNA-Seq Method

HFD pigs used in the experiment showed over 40% higher fat deposition in the lumbar location and over 30% higher FD according to average backfat thickness and peritoneal and loin fat than in LFD individuals. Analysis of FGL1 expression between high and LFD pigs showed that in 180-day-old animals, FGL1 was not differentially expressed (Table 1). Table 1 presents details of the phenotypic characteristics of HFD and LFD pigs.

### 3.2. Variant Calling and χ^2^ Test Analyses

The average number of detected raw reads per sample was 62,276,008, and after filtration, it was 61,480,178. Mapping to the pig reference genome (Sscrofa11.1 GCA_000003025.6 assembly) showed that 70.8% of uniquely mapped reads matched the annotated exon regions, and 16.5% matched the introns. Over 100,000 mutations were identified in analyzed pigs, of which over 7000 showed significant differences in genotype distribution between HFD and LFD groups. It was found that 73.8% of identified mutations belonged to known variants deposited in the NCBI database. The number of insertions and deletions constituted a mere 7%, and significance according to the χ^2^ test was 356. There were close to 2500 3′UTR significant variants. Significant downstream and intron mutations were numerous, over 2000 and 3500, respectively. Downstream and intron mutations are probably related to non-coding but functionally transcribed active genomic regions or unannotated exons (Table S2). However, they are considered an error or the result of poor genome annotation in most cases [25].

### 3.3. FGL1 Mutations Identified Using Variant Calling Analysis for 16 ZW Pigs

**Figure 1:**
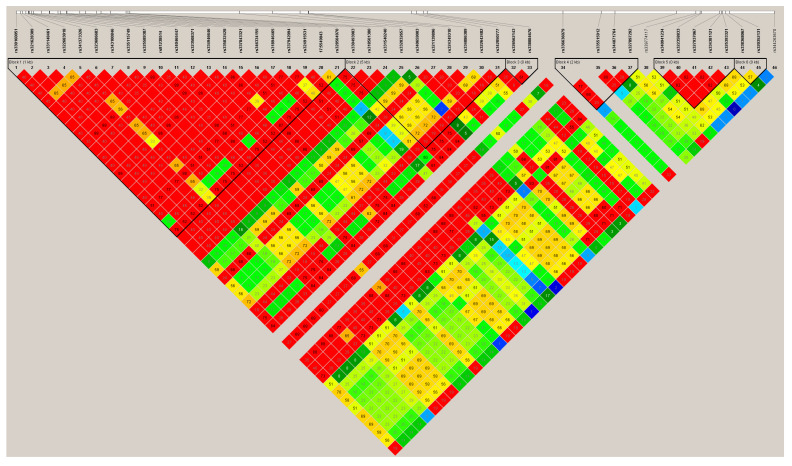
Linkage disequilibrium blocks estimated for SNPs identified across the FGL1 gene [23]. Red color shows high LD > 0.8, green and medium yellow 0.8 > LD > 0.5, and low blue LD < 0.5.

For further analysis, the *FGL1* variants were chosen because the *FGL1* plays an important role in the liver, the major organ for converting excess carbohydrates and proteins into fatty acids and triglycerides then exported and stored in adipose tissue [26]. On the other hand, String v. 11.0 showed that *FGL1* is often mentioned in publications as co-expressed (Figure S1) in the liver or plasma, with genes strongly associated with the regulation of fat deposition. We have identified 114 mutations in the *FGL1* gene region, including 3′UTR, intron, and synonymous variants, and the χ^2^ test found 54 (3′UTR and intron) mutations as significant revealing differences in genotype distribution between the analyzed FD pig groups. The lowest *chicq* value (0.000335463) for 10 mutations in the 3′UTR region was observed. Haplotype analysis and visualization of linkage disequilibrium, including all SNPs, showed that they were clustered in a few blocks (Figure 1).

Haplotype analysis after variant calling calculation pinpointed that 10 of 3′UTR variants, including indels: NC_010459.5:g.5548991-5548992del and NC_010459.5:g. 5549161insCAGCA were 100% linked. Results of the variant calling for liver tissue are available as an Excel file containing a few sheets at shorturl.at/joxBT.

miRBase analysis showed that identified *FGL1* 3′UTR mutations may affect miRNA binding sites previously reported as correlated with obesity, including those involved in the insulin signaling pathway, maturity-onset diabetes of the young, type II diabetes mellitus, and adipocytokine signaling pathway (Table S3).

**Figure 2:**
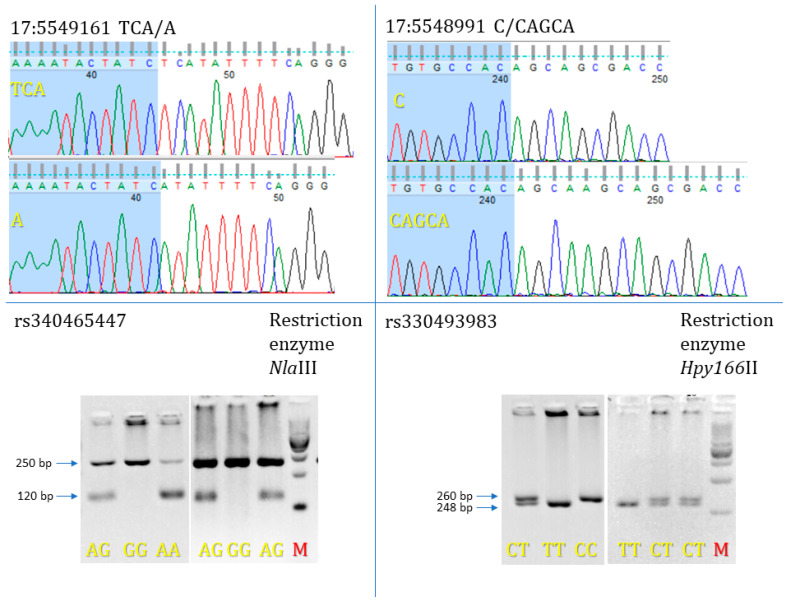
Showed genotyping results obtained for *FGL1* 3′UTR mutations two SNPs rs340465447, rs330493983 and two INDELs: NC_010459.5:g.5548991-5548992del and NC_010459.5:g.5549161insCAGCA. M—marker 100 bp DNA Ladder (New England Biolabs Inc., Ipswich, MA, USA). rs340465447 and rs330493983 SNPs were separated on 3.5% and 5% agarose gel, respectively.

Four of the identified 3′UTR *FGL1* mutations were chosen to evaluate their effect on pig phenotypes (SNPs: rs340465447 and rs330493983; indels: NC_010459.5:g.5548991-5548992del and NC_010459.5:g. 5549161insCAGCA). Figure 2 presents the results of *FGL1* mutation genotyping. New indel mutations were submitted as new alleles of the *FGL1* gene to GenBank under accession number MW827172.

Preliminary association analysis, including 16 pigs that differed in fat deposition (the same as in the RNA-seq method), showed that 10 fully linked 3′UTR polymorphisms were significantly associated with fat deposition. Although only two alternative homozygous animals were found (AA-rs340465447 or CC-rs330493983), the heterozygous animals were quite distinct in FD traits from reference homozygotes. They showed almost 1 cm thicker backfat (*p* < 0.001) at three measured points of lumbar, 800 and 730 g (*p* < 0.001) heavier loin and ham fats, respectively. The differences between the FGL1 genotypes are shown in Table 2.

### 3.4. GLM Analysis between Złotnicka White Phenotype and FGL1 Variants

The analysis of rs340465447, rs330493983, and indels NC_010459.5:g.5548991-5548992del (TCA/A) and NC_010459.5:g.5549161insCAGCA (C/CAGCA) FGL1 mutations of the 3′UTR region, including the more numerous Złotnicka White population (*n =* 72), showed that polymorphism linkage was not as strong as in the 16 individuals selected for RNA sequencing. Two SNPs were fully linked but revealed a slightly different allele distribution than observed for two indel mutations (Table S4). For NC_010459.5:g.5548991-5548992del(TCA/A), we identified a higher number of heterozygotes than for rs340465447 (G/A) and fewer alternate homozygotes. According to the SNPs, the ZW population was not in HWE.

In turn, indel frequency was consistent with the HWE. The association analysis was performed for rs340465447 (G/A) and NC_010459.5:g.5548991-5548992del (TCA/A) and showed that these SNP mutations are associated with IMF and subcutaneous fat deposition traits. Pigs with the AA genotype showed 24%, 23%, and 32% higher IMF, ABT, and loin fat, respectively. Additive effect analysis confirmed this observation and showed that a change A → G leads to −0.24 cm (*p* < 0.01) of ABT, +2.90 cm^2^ (*p* < 0.01) of loin eye area and +9 day (*p* < 0.05) of slaughter age. The rs340465447 mutation leads to changes in daily gain and pH of loin and ham values measured 24 h after slaughter (Table 3).

The NC_010459.5:g.5548991-5548992del (TCA/A) polymorphism influenced fat and meat traits, such as loin and ham mass, meat percentage, loin eye area and primary cuts, and different kinds of subcutaneous fats, such as average backfat thickness and loin and ham fats. In turn, for IMF, only a value of trend was observed. The dominance effect comparing heterozygous with homozygous values showed that changes ins/del → ins/ins leads to +2.04% (*p* < 0.01) of meat percentage, +280 g (*p* < 0.05) of ham mass and −0.32 cm (*p* < 0.01) in backfat thickness in the K1 point—over the lateral edge of the longissimus dorsi muscle (Table 4). The differences between pig traits were shown even between ins/del heterozygotes and ins/ins homozygotes.

### 3.5. FGL1 Frequency in Pigs Active in Polish Breeding

SNP and indel mutation frequencies were tested in the boars and sows used in Polish breeding. Analysis showed that rs340465447/rs330493983 FGL1 polymorphisms were rare in the PLW and Puławska breeds. There were only heterozygous variants present. In turn, in the PL and Duroc, alternate homozygous AA was also observed. Deletion NC_010459.5:g.5548991-5548992del (TCA/A) or insertion NC_010459.5:g. 5549161insCAGCA(C/CAGCA) showed a lower frequency. Only heterozygotes were observed in the PL, and none of the alternate homozygote pigs were found (Table S4).

## 4. Discussion

### 4.1. Pig Potential as an Animal Model in Fat Deposition Research

At the end of the 20th century and the beginning of the 21st century, the rate of life significantly accelerated due to vast civilization leap, the development of science, and access to new technologies. The consequence of increased stress levels resulted in civilization diseases, such as obesity leading to substantial health disorders such as diabetes [27], hypertension [28], cancer [29], or premature death [30]. In the face of such a severe epidemic, various studies have determined the precise processes related to fat deposition [31], treatment of its effects, and analysis of environmental factors conducive to obesity [32]. The introduction of appropriate prevention through changing nutritional habits has also been studied and implemented [33].

The molecular process associated with fat accumulation is often challenging in humans; the specific clinical and physiological conditions related to obesity may be limiting due to costs or complexity, the difficulties in obtaining appropriate tissue samples, or ethical problems. Thus, researchers use animal models where molecular DNA/RNA/protein functions remain unchanged between species. Pig animal model better reflects the processes occurring in humans than small lab animals, as they show human-like body proportions [34], metabolism [35], and a similar distribution of adipose tissue and adipocyte size [36]. On the other hand, mutations identified in pigs leading to significant phenotype disorders deliver the information that may be applied in human research. The identified mutations may be helpful in finding a potential problem solution. For example, the melanocortin receptor 4 (*MC4R)* gene, the mutation described by Kim et al. [37], was found to be significantly associated with food consumption and increasing the fat content in pigs in different pig breeds and countries, including in Poland [38]. A few years later, Chagnon et al. [39] found several mutations significantly associated with morbid obesity in the study of human *MC4R*.

The present study identified mutations in the *FGL1* gene, which were significantly related to fat deposition in Złotnicka White (ZW) pigs using variant calling methods based on RNA-seq data. We found more than 50 polymorphism sites that, according to the χ^2^ test, showed significantly different genotype/allele distribution when comparing low- and high-fat deposition pig groups. Most of them were located in the 3′UTR region, which controls gene expression and does not change the amino acid sequence. As previously mentioned, *FGL1* is expressed mainly in the liver and is related to the regeneration of this organ, being involved in the hepatocyte proliferation process [8]. Demchev et al. [9] suggested in their reports that a lack of the *FGL1* gene leads to increased body weight and an abnormal plasma lipid profile. Nevertheless, RNA-seq data used in the present study for gene variant identification showed that *FGL1* was not differentially expressed between high- and low-FD pig groups at 180 days old. However, these findings do not preclude prior differentiation at the beginning of ontogenetic growth or in an embryonic stage.

Moreover, our String v. 11.0 analysis showed that *FGL1* is often mentioned in the publications as co-expressed in the liver or plasma with apolipoprotein C3 (*APOC3*), apolipoprotein A1 (*APOA1*), and apolipoprotein A2 (*APOA2*), which are strongly associated with the regulation of very-low-density lipoproteins, cholesterol efflux, and transport, and triglyceride catabolic process with [40].

It is assumed that identified significant *FGL1* 3′UTR variants change binding sites for numerous miRNAs previously described as playing a role in regulating lipid processes related to diabetes, appearing as potentially regulating porcine *FGL1* gene expression, such as mir338-5p and mir338-3p. Their binding site was probably controlled by rs330493983 FGL1 SNP, which revealed a significant effect on fat deposition in pigs. The T variant of rs330493983 occurring in lean pigs favors miRNA-338 binding, whereas the C variant precluded interacting with this regulator. miR-338-3p is expressed in the liver and is related to cancer pathogenesis [41,42]. However, this miRNA was also examined for its role in metabolic disorders such as hepatic insulin resistance and mediation in glycogenesis by regulating the AKT/GSK3β signaling pathway. Moreover, Li et al. [41] showed that mir338-3p was down-regulated in db/db, HFD-fed, and TNFa-treated C57BL/6j mice. This observation suggested an adverse effect of mir-338-3p on fat deposition, similar to hepatic *FGL1*, which Demchev et al. [9] observed in *FGL1*-null mice. Thus, the binding site in the 3′UTR region significantly affecting FD in pigs indicates likely regulation of mir-338-3p-FGL1. However, it should be confirmed in further experimental research, including cell culture and luciferase assay.

### 4.2. FGL1 Variants as a Potential Selective Marker to Improve Fat Content in Pigs

Long-term selection toward lean meat in pigs, both in the breeds used as dam and sire lines, has led to decreased backfat thickness and IMF, which determine meat flavor and technological suitability [43]. The attempted recovery of higher fat content in pig carcasses by traditional breeding methods is time-consuming and expensive. The application of genetic markers enables the selection process acceleration due to the possibility of young animal evaluation and removing those with adverse gene variants. In Polish pig breeding, only one requirement is identifying a mutation in the *RYR1* gene and eliminating carriers and burdened individuals from the dam and sire populations. Moreover, the Polish Ministry of Agriculture and Rural Development presently funds the monitoring of *IGF2* and *MC4R* polymorphisms [37,44] in PL pigs as a maternal component to indicate the possibility of improving fat tissue levels. However, they still are searching for new potential genetic markers that could be useful in this case.

In the present study, the possibility of using *FGL1* 3′UTR variants was tested in the ZW breed, which is not under selection pressure, so within this population, a wide range of fat-related and meat traits is represented; thus, ZW is a suitable material for association study. Our research showed for interesting correlation of rs340465447_A, rs330493983_C, NC_010459.5:g.5548991-5548992del_A and NC_010459.5:g.5549161insCAGCA_CAGCA *FGL1* alleles that were positively associated with subcutaneous fat and also IMF. Identification of these alleles in Polish pig populations used as maternal and paternal components in breeding was carried out. Their presence in these populations was rather poor. However, in the PL and Duroc pigs, allele rs340465447_A occurred in the alternate homozygous form, which is promising if breeders would try to introduce this selection marker to increase fat levels, especially as only boars were analyzed in the PL and Duroc; therefore, its spread in the PL population would be easier.

## 5. Conclusions

Mice and human studies show that *FGL1* is indirectly related to the occurrence of obesity and diabetes. Our study supports this theory because we detected interesting *FGL1* variants in the 3′UTR region using liver RNA-seq data related to fat levels in the pig carcasses. We indicated that identified mutations are probably associated with miRNA binding, which regulates *FGL1* expression, and probably fat deposition in pigs. Moreover, association analysis in ZW pigs pinpointed the possibility of the *FGL1* variant, especially rs340465447_A utility, in the selection process toward increasing fat level in pigs since fat is the leading meat flavor carrier.

## Figures and Tables

**Table 1:** Characteristics of Zlotnicka White pigs representing high and low-fat deposition traits; means ± SD.

Traits	HFD (n = 8)			LFD (n = 8)					All Pigs (n = 72)	
	Mean		SD	Mean		SD	*p*-Value	Group Diff *	Mean	SD
Daily gain (g)	641	a	34.5	718	b	84.2	0.03	11%	700	106
Backfat thickness (cm)	2.09	A	0.31	1.53	B	0.36	0.004	27%	1.90	0.39
Peritoneal fat (kg)	0.72	A	0.11	0.46	B	0.06	0.0001	36%	0.61	0.16
Backfat thickness in the K1 point (cm)	2.23	A	0.37	1.44	B	0.20	0.0002	35%	2.00	0.52
Ham fat mass with skin (kg)	2.49	A	0.21	1.80	B	0.23	1.86 × 10^−5^	28%	2.15	0.37
Loin fat mass with skin (kg)	2.37	A	0.17	1.58	B	0.28	2.06 × 10^−5^	33%	2.03	0.48
Fat over shoulder thickness (cm)	2.85	A	0.27	2.11	B	0.31	0.0002	26%	2.75	0.61
Lumbar fat I thickness (cm)	2.43	A	0.26	1.44	B	0.32	1.096 × 10^−5^	41%	2.16	0.55
Lumbar fat II thickness (cm)	2.20	A	0.47	1.26	B	0.21	0.0004	43%	1.95	0.54
Lumbar fat III thickness (cm)	2.74	A	0.63	1.80	B	0.29	0.003	34%	2.40	0.59
Average backfat thickness (cm)	2.46	A	0.27	1.60	B	0.17	8.63 × 10^−6^	35%	2.27	0.45
*FGL1* expression level	3799.6		932.61	3731.6		1125.4	0.45			

Abbreviation: SD—standard deviation, HDF—high-fat deposition, LFD—low-fat deposition. * Group diff—differences between groups in percentage. Values with the same letters belong to the same statistical group (A, B = *p* < 0.01; a, b = *p* < 0.05).

**Table 2:** Means ± S.E. based on ANOVA test for important pig traits dependent on rs340465447, rs330493983, INDELs: NC_010459.5:g.5548991-5548992del and NC_010459.5:g.5549161insCAGCA genotypes in 16 ZW pigs differed in fat deposition.

Traits	*FGL1* Genotype		
	GG TT	AG CT	AA CC
Loin weight (kg)	4.87 ± 0.40 ^A^	4.38 ± 0.30 ^B^	4.39 ± 0.30
Ham weight (kg)	8.06 ± 0.30 ^a^	7.48 ± 0.30 ^b^	7.07 ± 0.7 ^b^
Feet mass (kg)	1.02 ± 0.05 ^Aa^	0.95 ± 0.01 ^b^	0.89 ± 0.07 ^Bc^
Knuckle fat with skin (kg)	0.21 ± 0.01 ^A^	0.25 ± 0.02 ^B^	0.24 ± 0.01 ^AB^
Loin eye height (cm)	6.80 ± 0.16 ^A^	6.22 ± 0.27 ^B^	6.45 ± 0.32 ^AB^
Loin eye area (cm^2^)	47.1 ± 2.75 ^a^	43.5 ± 3.45 ^a^	42.4 ± 3.48 ^ab^
Meat percentage in primary cuts (kg)	64.2 ± 1.08 ^A^	58.9 ± 1.21 ^Ba^	58.3 ± 2.54 ^b^
Meat percentage %	56.0 ± 1.05 ^A^	50.5 ± 1.21 ^Ba^	50.2 ± 2.51 ^b^
Peritoneal fat (kg)	0.46 ± 1.99 ^A^	0.70 ± 2.88 ^B^	0.76 ± 4.89 ^AB^
Loin fat with skin (kg)	1.58 ± 0.20 ^Aa^	2.39 ± 0.20 ^B^	2.28 ± 0.10 ^Bb^
Ham fat with skin (kg)	1.80 ± 0.30 ^A^	2.53 ± 0.01 ^B^	2.38 ± 0.02 ^B^
Backfat over shoulder (cm)	2.11 ± 0.31 ^A^	2.83 ± 0.10 ^B^	2.90 ± 0.31 ^B^
Backfat over back (cm)	1.53 ± 0.36 ^A^	2.10 ± 0.36 ^B^	2.05 ± 0.05 ^B^
Backfat over lumbar I	1.44 ± 0.32 ^Aa^	2.43 ± 0.28 ^B^	2.40 ± 0.20 ^b^
Backfat over lumbar II	1.26 ± 0.21 ^A^	2.18 ± 0.52 ^B^	2.25 ± 0.25 ^B^
Backfat over lumbar III	1.80 ± 0.71 ^Aa^	2.78 ± 0.20 ^B^	2.60 ± 0.29 ^b^
Average backfat thickness (cm)	1.63 ± 0.18 ^A^	2.47 ± 0.31 ^B^	2.44 ± 0.10 ^B^
Backfat in the point C1	1.44 ± 0.20 ^Aa^	2.25 ± 0.15 ^B^	2.15 ± 0.41 ^Bb^
Backfat in the point K1	1.44 ± 0.20 ^Aa^	2.25 ± 0.15 ^B^	2.15 ± 0.41 ^Bb^

Allele: G—wild, A—mutation. The probability was measured at least squares means for gene effect where Pr > |t| for H0: LSMean = LSMean (SAS v. 8.02). Values with the same superscripts belong to the same statistical group (A, B = *p* < 0.01; a, b = *p* < 0.05), *p*-value

**Table 3:** Least mean square (LSM) ± S.E. for important pig traits dependent on ENSSSCT00000037614.2:c.*1662G>A *FGL1* genotypes (rs340465447).

Traits	*FGL1* Genotype							GLM Significance		Additive Effect	Dominance Effect
	AA (n = 26)		AG (n = 16)		GG (n = 30)		*p*-Value	X2P	*FGL1*	A → G	Het → Hom
	LSM	SE	LSM	SE	LSM	SE					
pH_ham24_	5.57 ^AB^	0.03	5.64 ^A^	0.03	5.53 ^B^	0.02	0.0080	x	**	ns	−0.04 *
pH_loin24_	5.59 ^A^	0.02	5.55 ^AB^	0.03	5.46 ^B^	0.02	0.0006	x	***	−0.06 ***	ns
IMF	1.64 ^a^	0.10	1.32 ^b^	0.09	1.49 ^ab^	0.06	0.0462	x	*	ns	+0.13 *
Carcass yield (kg)	74.8 ^a^	0.18	74.6 ^ab^	0.22	74.1 ^b^	0.16	0.0262	***	*	ns	ns
Average backfat thickness (cm)	2.61 ^a^	0.14	2.28 ^ab^	0.13	2.12 ^b^	0.10	0.0234	ns	*	−0.24 **	ns
Loin eye height (cm)	5.62 ^A^	0.19	6.08 ^AB^	0.18	6.40 ^B^	0.13	0.0046	ns	**	+0.41 **	ns
Loin eye area (cm^2^)	40.1 ^a^	1.53	44.3 ^ab^	1.47	45.3 ^b^	1.10	0.0236	**	*	+2.90 **	ns
Meat percentage (%)	50.9	1.13	52.3	1.08	53.5	0.80	0.1491	ns	ns	+1.33 *	ns
Primary cut (kg)	18.8	0.43	19.3	0.41	19.8	0.31	0.1919	ns	ns	+0.60 *	ns
Loin fat with skin (kg)	2.32 ^A^	0.13	2.17 ^ABa^	0.13	1.76 ^Bb^	0.09	^AB^ 0.0033 ^ab^ 0.0344	***	**	−0.25 *	ns
Backfat over back (cm)	2.35	0.16	1.90	0.15	1.87	0.12	0.0557	ns	*	−0.23 *	ns
Backfat over lumbar I (cm)	2.61 ^A^	0.17	2.20 ^AB^	0.16	1.97 ^B^	0.12	0.0087	ns	**	−0.32 **	ns
Backfat over lumbar II (cm)	2.33 ^a^	0.16	1.94 ^ab^	0.15	1.76 ^b^	0.11	0.0136	ns	**	−0.28 **	ns
Backfat over lumbar III (cm)	2.72	0.18	2.52	0.17	2.24	0.13	0.0939	ns	*	−0.24 *	ns
Daily gain (0–180 days) kg	530	16	521	15	493	11	0.1529	ns	ns	−18 *	ns
Slaughter age (day)	187	6.4	192	6.2	204	4.9	0.0831	x	*	+9 *	ns

Allele: G—wild, A—mutation. Mean and SE were estimated using GLM model, values with the same superscripts belong to the same statistical group (A, B = *p* < 0.01; a, b = *p* < 0.05), p-value in GLM significant * *p* < 0.05, ** *p* < 0.01, *** *p* < 0.001, ns—not significant, X2P—covariate for weight of the right side of the carcass.

**Table 4:** Least mean square (LSM) ± S.E. for important pig traits dependent on NC_010459.5:g.5548991-5548992del deletion (TCA/A).

Traits	*FGL1* Genotype							GLM Significance		Additive Effect	Dominance Effect
	TCA/TCA (n = 31)		TCA/A (n = 32)		A/A (n = 9)		*p*-Value	X2P	FGL1	Ins → Del	Het → Hom
	LSM	SE	LSM	SE	LSM	SE					
IMF	1.49	0.06	1.40	0.06	1.90	0.21	0.0760	x	*	0.21 *	0.15 *
pH_ham24_	5.53 ^a^	0.02	5.62 ^b^	0.02	5.58 ^ab^	0.06	0.0354	x	**	ns	ns
pH_loin24_	5.47 ^a^	0.02	5.56 ^b^	0.02	5.60 ^ab^	0.04	0.0103	x	**	0.06 *	ns
Carcass yield (kg)	74.08 ^A^	0.14	74.6 ^B^	0.16	75.4 ^AB^	0.35	0.0039	***	**	ns	ns
Average backfat thickness (cm)	2.11 ^A^	0.10	2.56 ^B^	0.10	1.99 ^AB^	0.24	0.0093	ns	**	ns	−0.26 **
Loin eye area (cm^2^)	45.5 *	1.10	41.6	1.20	43.7	2.70	0.0506	**	*	ns	ns
Loin eye height (cm)	6.42 ^a^	0.14	5.81 ^b^	0.15	5.82 ^ab^	0.33	0.0107	ns	**	−0.32 *	ns
Meat percentage (%)	53.6 ^a^	0.74	50.6 ^b^	0.80	55.8 ^ab^	1.81	0.0205	ns	**	ns	+2.04 **
Primary cut (kg)	19.8 ^a^	0.28	18.6 ^b^	0.31	20.7 ^ab^	0.69	0.0227	***	**	ns	+0.70 *
Loin fat with skin (kg)	1.76 ^A^	0.09	2.33 ^B^	0.10	1.88 ^AB^	0.22	0.0003	***	***	ns	−0.28 **
Loin mass without fat and skin (kg)	4.62 ^a^	0.10	4.24 ^b^	0.10	4.82 ^ab^	0.24	0.0326	***	**	ns	+0.22 *
Ham fat mass (kg)	1.99 ^a^	0.08	2.30 ^b^	0.09	1.84 ^ab^	0.19	0.0271	*	**	ns	−0.21 **
Ham mass (kg)	7.75 ^a^	0.14	7.31 ^b^	0.15	8.16 ^ab^	0.26	0.0374	***	**	ns	+0.28 *
Backfat over back (cm)	1.85	0.11	2.23	0.12	1.72	0.3	0.0827	ns	*	ns	−0.22 *
Backfat over lumbar I (cm)	1.97 ^a^	0.10	2.50 ^b^	0.10	2.02 ^ab^	0.29	0.0102	ns	*	ns	−0.25 **
Backfat over lumbar II (cm)	1.75 ^A^	0.11	2.25 ^B^	0.12	1.65 ^AB^	0.26	0.0078	ns	**	ns	−0.28 **
Backfat over lumbar III (cm)	2.23 ^A^	0.12	2.78 ^B^	0.13	2.03 ^AB^	0.29	0.0083	ns	**	ns	−0.33 **
Backfat in the point C1	1.86 ^a^	0.12	2.38 ^b^	0.13	1.55 ^ab^	0.29	0.0130	ns	**	ns	−0.34 **
Backfat in the point K1	1.85 ^a^	0.11	2.38 ^b^	0.13	1.60 ^ab^	0.28	0.0118	ns	**	ns	−0.32 **
Daily gain	729	20	662	22	714	50	0.0754	ns	*	ns	+30 *

Allele: TCA—wild, A—mutation. Mean and SE were estimated using the GLM model; values with the same superscripts belong to the same statistical group (A, B = *p* < 0.01; a, b = *p* < 0.05), *p*-value in GLM significant * *p* < 0.05, ** *p* < 0.01, *** *p* < 0.001, * in FGL1 genotypes showed trends, ns—not significant, K1 point—backfat thickness over the lateral edge of longissimus dorsi muscle, C1—backfat thickness in height extension of loin eye, X2P—covariate for weight of the right side of the carcass.

## Data Availability

New INDEL mutations identified in the FGL1 gene were submitted to GenBank with access number MW827172 (NCBI database). The sequence data for RNA libraries have been submitted to the Gene Expression Omnibus (GSE160436). Variant calling results are available at link shorturl.at/ksKU1 (accessed on 20 Oct 2021).

## Supplementary Materials

The following supporting information can be downloaded at: https://www.mdpi.com/article/10.3390/genes13081419/s1. Table S1: Primers used in the study. Table S2: Potential changes in miRNA binding sites identified by miRBase dependent on mutations in 3′UTR region of FGL1 gene in pigs Table S3: Means ± S.E. based on ANOVA test for important pig traits dependent on rs340465447, rs330493983, INDELs: NC_010459.5:g.5548991-5548992del and NC_010459.5:g.5549161insCAGCA genotypes in 16 ZW pigs differed in fat deposition. Table S4: The frequencies of alleles and genotypes of SNP and INDEL in 3′UTR of FGL1 gene in Złotnicka White and pigs active in Polish breeding. Figure S1: Fgl1 and their interactions. By color are indicated the involvement of particular interactions in the biological process, molecular function and KEGG pathway associated with lipid metabolism. The protein interactions were generated by STRINGv.11b: functional protein association networks. https://string-db.org/.
