# Supplementary material for: Variations in Fibrinogen-like 1 (FGL1) Gene Locus as a Genetic Marker Related to Fat Deposition Based on Pig Model and Liver RNA-Seq Data
Source: Genes (Basel). 2022 Aug 9;13(8):1419. doi: 10.3390/genes13081419 (PMC9407393; doi:10.3390/genes13081419)
Supplement: Supplementary file 1 [file genes-13-01419-s001.zip › suplement file.pdf]

Table S1. Primers used in study

Gene	Primers	Method	Restriction enzyme
rs330493983	F- TTGGAATGTTCACATGTGTGTTTA	PCR-ACRS	*Hpy166*II
	R-ATCTATGCTATGTTATCTCCCCAGTC	DNA sequencing	
	F-TGGGCTTTCATTTCAGCAG		
	R-TTAAGTGATAAAGGCAGTTAAGAACG		
rs340465447		PCR-RFLP, DNA sequencing	*Nla*III
	F-GGCAATATGGCTTAGTGATCC		
	R-TGAAGTAGGCATTAAGGGACAA		
17:5548991	F-TGAACACAGGTCTCTGACTGC		
17:5549161	R-AAAGCTAATAAGTAGGGGAACAAGG	DNA sequencing	

Table S2. Characteristic of porcine liver transcriptome in the context of identified mutations

Mutation (variant)	Quantity	
	total	$\chi2$ test*
Total	103.792	7249
Know variant % (having rs number)	73.8	82.2
Analyzed transcript	14152	3322
Heterosygosity %	42.5	-
Insertion	3.648	202
Deletion	3.818	154
SNP	96.325	6.893
High effect	3927	70
Low effect	12.854	998
Moderate effect	5.250	376
Missense variant	5.073	371
Silent variant	11.241	914
3′UTR	32.574	2.403
5′UTR premature start codon	776	52
5′UTR	4.980	305
Frameshift variant	1.596	45
Splice region variant	3.267	91
Inframe variant	436	4
Downstream gene	31.327	2.100
Upstream gene	15.830	933
Intergenic region	9.349	689
Intron	52.409	3.537
Start lost	11	1
Stop gain	48	3
Stop lost	36	1

Abbreviation: * significant differences based on $\chi2$ test in genotype distribution between HFD and LFD groups. High, moderate and low effects - it is power of effect on protein expression, amino acid sequence and phenotype. Definition of high, low and moderate effects is given at link http://snpeff.sourceforge.net/SnpEff_manual.html

Table S3. Potential changes in miRNA binding sites identified by miRBase dependent on mutations in 3'UTR region of *FGL1* gene in pigs

FGL1 3'UTR mutation	Mutation type	miRNA binding
**NC_010459.5:g.17:5548991**	G/TGCTG	eca-miR-8979, gga-miR-6714-3p, gga-miR-1637, bdi-miR7787-3p
**NC_010459.5:g.17:5549161**	TGA/A	cel-miR-84-5p, ptc-miR478h, ptc-miR478i, ptc-miR478j, ptc-miR478k, ptc-miR478l, ptc-miR478m, ptc-miR478o, ptc-miR478q, ptc-miR478r, ptc-miR478s, ptc-miR478n, gra-miR7584e, bmo-miR-2769
rs330160051	A/G	bmo-miR-2843-2-3p, cfa-miR-8839, mmu-miR-7004-5p, gga-miR-12251-3p, mmu-miR-6918-3p, ipu-miR-7563c, mdo-miR-1545f-3p
rs323686683	T/A	gra-miR8661, dme-miR-969-5p, dsi-miR-969-5p, mmu-miR-7078-3p, hsa-miR-4661-5p
rs343100846	A/G	emu-miR-10-3p, pma-miR-4602, gga-miR-6605-5p, egr-miR-10a-3p, mmu-miR-375-5p, rno-miR-375-5p, hsa-miR-375-5p, pal-miR-375-5p, cpo-miR-375-5p, dno-miR-375-5p, ocu-miR-375-5p
rs335115749	T/C	tca-miR-3867-5p, osa-miR5819
**rs340465447**	C/T	gra-miR8705, pal-miR-7140-3p, mmu-miR-6715-5p, pal-miR-7140-5p, bmo-miR-3309
rs337843321	G/T	hpo-miR-72-3p, cpi-miR-9589-5p
rs346334155	G/A	hco-miR-5958
**rs330493983**	G/A	mmu-miR-489-5p, mmu-miR-669a-3-3p, ame-miR-190-5p, ppc-miR-8356a-3p, **rno-miR-338-3p, mdo-miR-338, cfa-miR-338, bta-miR-338, mmu-miR-3065-5p, hsa-miR-3065-5p, rno-miR-3065-5p, cgr-miR-338, ggo-miR-338, oha-miR-338-3p, gga-miR-338-3p, cpi-miR-338a-3p, cli-miR-338b-3p, pbv-miR-338-3p, pal-miR-338-3p**, bta-miR-3065, **cpo-miR-338-3p, dno-miR-338-3p**
rs318501380	T/C	aga-miR-10374-3p, bdi-miR5057, hco-miR-5971, sme-miR-1c-5p, str-miR-8374-5p lca-miR-15a, ppa-miR-15b, sla-miR-15a
rs331540240	T/G	aca-miR-1677b-5p, oha-miR-1677b-5p, pbv-miR-1677-5p, abu-miR-24c, **hsa-miR-4686**
rs337942094	T/C	**emu-miR-31-3p**, mdo-miR-12407-3p, oni-miR-10707, bta-miR-431, eca-miR-431 eca-miR-9173, ssa-miR-730a-2-3p, ssa-miR-730a-3p
rs328504970	T/A	mse-miR-6095, bma-miR-9537
rs341373326	A/C	pab-miR11509, dme-miR-969-5p, dsi-miR-969-5p, pla-miR11608
rs321628309	G/T	sma-miR-8450-5p, sma-miR-8484-3p, mml-miR-3167-5p, rno-miR-544-5p, **gsa-miR-184b-5p**, dsi-miR-972-5p, hsa-miR-8076, cin-miR-4006f-3p
rs331148461	C/G	hsa-miR-6876-3p, eel-miR-11054-3p, mml-miR-892c-3p, gma-miR9736, ppc-miR-8304a-3p, hsa-miR-892c-3p
rs322683018	C/T	hsa-miR-6875-5p, lja-miR11089c-5p
rs325589387	G/C	gma-miR4367, stu-miR8048-5p, **hsa-miR-4780**, ath-miR4240, eun-miR159-5p, **rno-miR-345-5p, mmu-miR-345-5p**, atr-miR8585
rs334946646	G/A	stu-miR8028-5p, **hsa-miR-146b-5p, rno-miR-146b-5p, bta-miR-146b, cgr-miR-146b-5p, ggo-miR-146b, chi-miR-146b-5p, cja-miR-146b, pal-miR-146b-5p, dno-miR-146b-5p, ppa-miR-146b, mmr-miR-146b, nle-miR-146b, sbo-miR-146b, pha-miR-146b,ocu-miR-146b-5p**
rs324915531	C/T	oan-miR-1421b-5p, hsa-miR-597-3p, gga-miR-7448-3p, sfr-miR-279c-5p, oan-miR-1421x-5p, nve-miR-2032a-3p, pte-miR-278b-5p
rs81238514	A/G	**egr-miR-190-5p, emu-miR-190-5p, fhe-miR-190, mco-miR-190-3p**
rs342038820	T/C	sly-miR9473-5p, oni-miR-10929, **hsa-miR-511-5p, mml-miR-511-5p, ptr-miR-511, hsa-miR-3133**, **ppy-miR-511, cpo-miR-511-5p**, mmu-miR-701-3p
rs710773157	-/GGAAAT	gma-miR9764, hpo-miR-10050-5p
NC_010459.5:g.17:5549043	A/G	**dno-miR-338-5p**, dse-miR-9685-3p
rs342038820	T/C	sly-miR9473-5p, oni-miR-10929, **hsa-miR-511-5p, mml-miR-511-5p, ptr-miR-511 hsa-miR-3133, ppy-miR-511, cpo-miR-511-5p,** mmu-miR-701-3p
NC_010459.5:g.17:5549245	T/AGT	mdo-miR-12348-5p, gga-miR-1667-5p, bma-miR-5855
rs703172161	G/T	sly-miR9473-5p, **hsa-miR-3133**, gsa-miR-8-3p
rs1110718994	CT/-	cbr-miR-7580, hpo-miR-10043d-5p, nle-miR-4670, oan-miR-1422e-5p, **ssa-miR-26b-3p**, vvi-miR3638-3p

Sequences were search for mature miRNA, E-value cut-off=10. Bold mutation were included in to association analysis in ZW pigs. **Bolded mutation** were subsequently used in the association study. **Bolded miRNA** are involved in the lipid metabolism changes and obesity, inducing: Insulin signaling pathway(hsa04910) - miR-146b-5p, miR-26b-3p, miR-3065-5p, miR-3133, miR-31-3p, miR-338-3p, miR-338-5p, miR-345-5p, miR-4686, miR-4780, miR-511; Maturity onset diabetes of the young (hsa04950) - miR-26b-3p, miR-3065-5p, miR-3133, miR-338-3p, miR-338-5p, miR-345-5p, miR-511; Type II diabetes mellitus (hsa04930) - miR-146b-5p, miR-26b-3p, miR-3065-5p, miR-3133, miR-31-3p, miR-338-3p, miR-338-5p, miR-345-5p, miR-4686, miR-4780, miR-511; Adipocytokine signaling pathway (hsa04920) - miR-3065-5p, miR-3133, miR-338-3p, miR-338-5p, miR-4686, miR-511

Table S4. The frequencies of alleles and genotypes of SNP and INDEL in 3'UTR of *FGL1* gene in Zlotnicka White and pigs active in Polish breeding

Pig Breed	Frequencies of rs340465447 (G/A)						Frequencies of NC_010459.5:g.5548991-5548992del deletion (TCA/A)					
	Genotype			Allele		HWE* (*P*-value)	Genotype			Allele		HWE* (P-value)
	AA	AG	GG	A	G		TCA/TCA	TCA/A	A/A	TCA	A	
Zlotnicka White	0.36 (26)	0.22(16)	0.42 (30)	0.47	0.53	0.000003	0.43(31)	0.44 (32)	0.13 (9)	0.65	0.35	0.637167
Polish Large White	0 (0)	0.01 (3)	0.99 (205)	0.01	0.99	0.916565	1 (208)	0.0 (0)	0.0 (0)	1	0	-
Polish Landrace	0.02 (7)	0.09 (26)	0.89 (259)	0.14	0.86	0.000000	0.96 (280)	0.04 (12)	0.00 (0)	0.98	0.02	0.919542
Pulawska	0.0 (0)	0.03 (5)	0.97 (173)	0.01	0.99	0.849268	1(178)	0 (0)	0.00 (0)	1	0	-
Duroc	0.14 (10)	0.17 (12)	0.69 (50)	0.22	0.88	0.000011	1 (72)	0 (0)	0 (0)	1	0	-
Total	0.05 (43)	0.08 (62)	0.87 (717)	0.87	0.13	0.000000	0.94 (769)	0.05 (44)	0.01 (9)	0.87	0.13	0.000000

*HWE – Hardy-Weinberg Equilibrium: If p-value < 0.05 - not consistent with HWE, Not accurate if <5 individuals in any genotype group. In brackets number of individuals in the genotype group. Both SNPs rs340465447 (G/A) and rs330493983 (T/C) were 100% linked in the analysed individuals and both INDEL variants (NC_010459.5:g.5548991-5548992del deletion or insertion NC_010459.5:g. 5549161insCAGCA).

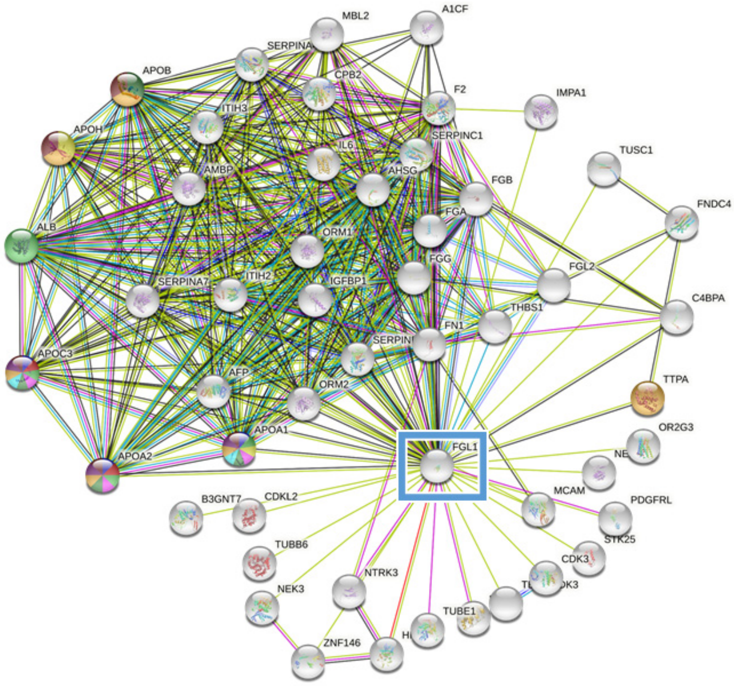

Biological process					
GOterm	description	Count in network	strength	FDR	marker
GO:0010903	Negative regulation of very-low density lipoprotein particle remodeling	3 of 3	2.58	8.79e-06	
GO:0060621	Negative regulation of cholesterol import	2 of 2	2.58	0.00045	
GO:0034375	High-density lipoprotein particle remodeling	4 of 17	1.96	6.36e-06	
GO:0051006	Positive regulation of lipoprotein lipase activity	2 of 9	1.93	0.0026	
GO:0043691	Reverse cholestarol transport	3 of 17	1.83	0.00023	
GO:0033344	cholesterol efflux	4 of 24	1.81	1.91e-05	
GO:0010896	Regulation of triglyceride catabolic process	2 of 12	1.81	0.0037	
GO:0006869	Lipid transport	6 of 272	0.93	0.00078	
**Molecular function**					
GO:0070653	High-density lipoprotein particle receptor binding	3 of 3	2.58	8.14e-06	
**KEGG Pathway**					
hsa04979	Cholesterol metabolism	5 of 48	1.6	9.13e-06	

Figure S1. Fgl1 and their interactions. By color are indicated the involvement of particular interactions in the biological process, molecular function and KEGG pathway associated with lipid metabolism. The protein interactions were generated by STRING: functional protein association networks. https://string-db.org/
